# Effects of Tung Oil Composite Regenerating Agent on Rheological Properties and Microstructures of Reclaimed Asphalt Binder

**DOI:** 10.3390/ma15093197

**Published:** 2022-04-28

**Authors:** Qimin Wang, Qunshan Ye, Junhui Luo, Cheng Xie, Haobin Liu, Jianhua Liu, Mengnan Qin

**Affiliations:** 1 Guangxi Beitou Transportation Maintenance Technology Group Co., Ltd., Nanning 530201, China; wangqm1615@163.com (Q.W.); jhluo85@hotmail.com (J.L.); xiecheng1357@163.com (C.X.); liuhaobin9203@163.com (H.L.); 2 Key Laboratory of Road Structure and Material of Ministry of Transport (Changsha), Changsha University of Science and Technology, Changsha 410114, China; 3 Department of Traffic and Transportation Engineering, Changsha University of Science and Technology, Changsha 410114, China; 005848@csust.edu.cn (J.L.); szct@stu.csust.edu.cn (M.Q.)

**Keywords:** tung oil composite regenerating agent, aged asphalt, reclaimed asphalt, rheological properties, microstructure and morphology

## Abstract

The single light oil regenerating agent has certain limitations on the performance recovery of aged asphalt. In this study, tung oil, dioctyl phthalate (DOP), C9 petroleum resin, and organic montmorillonite (OMMT) were used to prepare the composite regenerating agent, and its optimal mix proportion was determined by the orthogonal experimental design. The rheological properties and anti-aging performance of reclaimed asphalt were studied by the dynamic shear rheometer (DSR) and bending beam rheometer (BBR); and the Fourier transform infrared (FTIR) spectrometer, gel permeation chromatography (GPC), and scanning electron microscope (SEM) were adopted to explore its microstructure, morphology, and mechanism of action. The results show that with the addition of tung oil composite regenerating agent, the rheological properties of aged asphalt can be effectively recovered, even better than that of base asphalt. By using the complex modulus aging index (CMAI) and phase angle aging index (PMAI) it is found that the anti-aging performance of reclaimed asphalt is better than that of base asphalt. With the optimal content of the tung oil composite regenerating agent, the contents of characteristic functional groups and macromolecular asphaltenes in the aged asphalt can be reduced, indicating that the composite regenerating agent is beneficial to the dispersion and dissolution of polar substances in the aged asphalt. After aging, a large number of wrinkles appear on the surface of the asphalt. However, the addition of the tung oil composite regenerating agent can make the asphalt surface smooth, which indicates that the tung oil composite regenerating agent can restore the microstructure and morphology of aged asphalt to a certain extent.

## 1. Introduction

With the rapid development of road construction in China, asphalt pavement is widely used due to its excellent road performance. However, under the comprehensive action of various factors, such as a complex traffic environment and driving load, the aging phenomena of asphalt materials inevitably appear, which weakens the adhesion, aging resistance, low-temperature crack resistance, and other properties of asphalt pavement, eventually producing a large amount reclaimed asphalt pavement (RAP) [1,2]. By rationally treating a large number of old materials, the shortage of raw asphalts can be alleviated and an economical and environmentally friendly society with green transportation can be realized. Therefore, much attention has been paid to the recycling technology of reclaimed asphalt mixtures.

The recycling technology of asphalt is that a certain proportion of a regenerating agent is added to the reclaimed asphalt and effectively achieves its regeneration, thereby prolonging the service life of asphalt pavement [3,4,5]. The demand for regenerating agents is increasing day by day. However, it is difficult for the traditional single light oil to be used as the regenerating agent to achieve the ideal regeneration effect. In addition, the regeneration cost is growing higher and higher, which restricts the recycling technology. Therefore, it is inevitable to develop a green, environmentally friendly, and economical composite regenerating agent to replace the traditional regenerating agent and single light oil.

At present, regenerants with mineral oil as the main component are widely used. However, its shortcomings such as strong volatility and non-renewable restrict the development of regeneration technology [6]. The reclaimed light oil is usually used as a regenerating agent because of its good performance, low cost, and easily available raw materials. For example, reclaimed vegetable oil, organic oil, aromatic extract, distilled tall oil, and bio-oil can be used as regenerating agents to restore the performance of aged asphalt [7,8,9]. The reclaimed cooking oil (WCO) is conducive to the physical properties, rheological properties, and other pavement behaviors of asphalt binders [10,11,12]. Eleyedath et al. [13] believed that the light components contained in such regenerating agents had a small molecular weight and low viscosity, which could quickly restore their performance after mixing with the aged asphalt; however, the difference in molecular weight between these lightweight components and other molecules was too large, which led to poor compatibility, and the rapid loss of small and medium molecules caused the poor durability of asphalt pavement. The use of bio-oil can improve the low-temperature cracking resistance of aged asphalt, and its microstructure is similar to that of base asphalt [14,15,16]. The residual soybean oil selected as a regenerating agent increases the permeability and reduces the viscosity, which is unfavorable for the high-temperature rutting resistance [17,18]. Moreover, the reclaimed engine oil chosen as a regenerating agent improves the viscoelasticity and flexibility of the asphalt binder, and its anti-stripping performance is comparable to that of base asphalt [19,20]. Zhang et al. [21] used reclaimed wood-derived bio-oil to balance the chemical components of aged asphalt. Tung oil with the main chemical component of fatty acid triglycerides can be used as a natural light oil to supplement and balance the missing components of aged asphalt [22].

From the above-mentioned information, most of the current research focuses on a single oil regenerating agent. However, a single light oil or other aromatic compounds used as a regenerating agent can only improve the fluidity of the asphalt but finds it difficult to restore or improve the overall performance of the reclaimed asphalt. In this paper, tung oil, dioctyl phthalate (DOP), C9 petroleum resin, and organic montmorillonite (OMMT) were compounded to prepare a composite regenerating agent of asphalt. The optimal content of each raw material of the composite regenerating agent was determined by the orthogonal design test, which effectively restored the rheological properties and anti-aging performance of aged asphalt, and combined with the microstructure test, its regeneration mechanism was analyzed as well.

## 2. Raw Materials and Test Methods

### 2.1. Raw Materials

The tung oil from a tung oil factory in Mianyang, Sichuan was used as the base oil; DOP from a chemical plant in Yixing, Wuxi was adopted as the plasticizer; C9 petroleum resin from a chemical plant in Dongguan, Guangdong was chosen as the tackifier resin; and OMMT with the advantages of barrier properties, aging resistance, and flame retardancy was selected from a mineral products processing plant in Hebei. The main technical indexes of each raw material are shown in Table 1.

### 2.2. Asphalt Preparation

#### 2.2.1. Aged Asphalt

The PG64-22 petroleum asphalt provided by Hunan Baoli International was used as the base asphalt, and the aged asphalt was prepared by the laboratory simulation accelerated aging test method. The specific operation is that the base asphalt is aged by the rolling thin film oven test (RTFOT) for 85 min, and then placed in a pressurized aging vessel (PAV) to accelerate the aging for 20 h. The technical indexes of base asphalt and aged asphalt are shown in Table 2.

#### 2.2.2. Reclaimed Asphalt

According to the previous research [22,23], for long-term-aged asphalt with RTFOT aging for 85 min and PAV aging for 20 h, the appropriate content of tung oil is in the range of 2–8%. We must consider that different sources of asphalts have different performances to some extent. The tung oil composite regenerating agent was blended with the aged asphalt (4%, 6%, 8%, 10%, and 12%) to prepare the reclaimed asphalt. The aged asphalt was kept at 135 ± 5 °C with the tung oil composite regenerating agent added. It was sheared for 20 min (3000 r/min) by the high-speed shear, and then continuously stirred for 10 min (500 r/min). The reclaimed asphalt is named according to the content of the regenerating agent, such as R-4% reclaimed asphalt, that is, the content of the tung oil composite regenerating agent in the reclaimed asphalt is 4%.

### 2.3. Test Methods

#### 2.3.1. Rheological Property Test

The DSR test can measure the complex modulus ($G^{*}$) and the phase angle (δ) of the asphalt, and the rutting resistance of asphalt pavement can be characterized by the rutting factor$\;G^{*}/\sin\delta$. In this study, high-temperature rheological properties were evaluated by temperature sweep and frequency sweep tests. A rotor with a diameter of 25 mm was selected for base asphalt and reclaimed asphalt, and its setting interval is 1 mm. However, an 8 mm rotor with an interval of 2 mm was chosen for the aged asphalt. The temperature range of the temperature sweep test is 42~72 °C. The temperatures of the frequency sweep are 28 °C, 40 °C, 52 °C, 64 °C, and 76 °C, and its frequency is in the range of 0.1–10 Hz at each temperature. The master curves of the complex modulus and phase angle were constructed by the time-temperature equivalence principle. Based on the time–temperature equivalence principle, the effect of the temperature and loading frequency on the asphalt material was converted into a reduced frequency by using the displacement factor. The displacement factor can be obtained by the WLF empirical equation:

(1) $$Log\alpha_{T}=\frac{-C_{1}\left(T-T_{ref}\right)}{C_{2}+T-T_{ref}}$$

where$\;\alpha_{T}\;$is the displacement factor at T; T_R_ is the reference temperature; and $C_{1}$ and $C_{2}$ are empirical constants.

In addition, the low temperature cracking resistance of asphalt can be characterized by the BBR test. The BBR test can measure the creep stiffness (*S*) and creep rate (*m*), and the test temperature includes −12 °C, −18 °C, and −24 °C.

#### 2.3.2. Anti-Aging Performance Test

The aging resistance of reclaimed asphalt was analyzed by rheological properties after PAV aging and UV aging. The aging resistance of reclaimed asphalt was analyzed according to the effects of rheological indicators (CMAI, PMAI) on the rheological properties of aged asphalt. The calculation of Formulas (2) and (3) is shown below:

(2) $$\mathrm{CMAI}=\frac{G^{*}}{G_{0}^{*}}$$

(3) $$\mathrm{PMAI}=\frac{\delta }{\delta_{0}}$$

where$\;G^{*}\;$is the complex modulus of asphalt after aging; $G_{0}^{*}\;$is the complex modulus of asphalt before aging; δ is the phase angle of asphalt after aging; and $\delta_{0}\;$is the phase angle of asphalt before aging.

#### 2.3.3. Micro Performance Test

Scanning electron microscopy (SEM) was used to compare the difference between the microstructure and morphology of reclaimed asphalt with the optimum content and base asphalt. The asphalt samples were sprayed with gold prior to SEM.

Fourier transform infrared (FTIR) spectroscopy was adopted to compare the differences in the composition of characteristic functional groups between the reclaimed asphalt with the optimum content and the base asphalt. FTIR spectroscopy studied the physical and chemical changes during the aging and regeneration processes of asphalt, and the information on chemical bonds or functional groups was obtained through the absorption peaks with FTIR spectroscopy. The changes in asphalt functional groups after adding the tung oil composite regenerating agent were analyzed. The FTIR test wavelength range is 500–4000 cm^−1^, and the number of scans is 32.

Gel permeation chromatography (GPC) analyzed the molecular weight distribution changes of asphalt during the aging and regeneration processes. The molecular weight distribution of asphalt measured by GPC is closely related to the macroscopic properties of asphalt [24]. In this study, the mobile phase is tetrahydrofuran (THF), the concentration of the asphalt sample is 2 mg/mL, and the flow rate is 10 mL/min.

## 3. Orthogonal Test Design and Analysis

We used orthogonal tables to analyze multi-factor and multi-level experiments [25]. Under the condition that the orthogonal test can ensure the level of each test factor, the same number of tests can simplify the test groups and improve the test efficiency. The components of the tung oil composite regenerating agent mainly composed of tung oil, DOP, C9 petroleum resin, and OMMT were taken as the main factors, and the factor levels of the orthogonal test design are shown in Table 3.

## 4. Test Results and Analysis

### 4.1. Orthogonal Test Results of Tung Oil Composite Regenerating Agent

In order to select the primary and secondary effects of each factor on each index, a 25 °C penetration and softening point, a 15 °C ductility, and 135 °C viscosity of reclaimed asphalt were used as evaluation indexes to discuss, analyze, and determine the optimal level of each factor. The orthogonal test results and preferred combinations are shown in Table 4 and Table 5, respectively.

It can be seen from Table 4 and Table 5 that the performance of aged asphalt can be restored by selecting 70% or 75% of tung oil; however, when the content of tung oil increases from 70% to 75%, the change trend of the penetration is relatively small, while the change trend of the ductility, viscosity, and softening are relatively large. Therefore, the optimal content of tung oil is 75%. With the increase in DOP content, the indexes of the softening point and viscosity first decrease and then increase, while the indexes of the penetration and ductility first increase and then decrease, indicating that the DOP starts to have adverse effects after improving the aging asphalt maximally. As a result, the optimal content of DOP is 15%. To meet the principle whereby the softening point is the minimum while the penetration and ductility are the maximum, the optimal contents of the C9 petroleum resin and OMMT are 6% and 9%, respectively.

According to the comprehensive analysis of the orthogonal test, the best combination of the tung oil composite regenerating agent is A_1_B_2_C_3_D_3_, namely tung oil: DOP: C9 petroleum resin: OMMT = 25:5:2:3.

### 4.2. Effects of Composite Regenerating Agent on the Rheological Properties of Reclaimed Asphalt

#### 4.2.1. Complex Modulus

**Figure 1:**
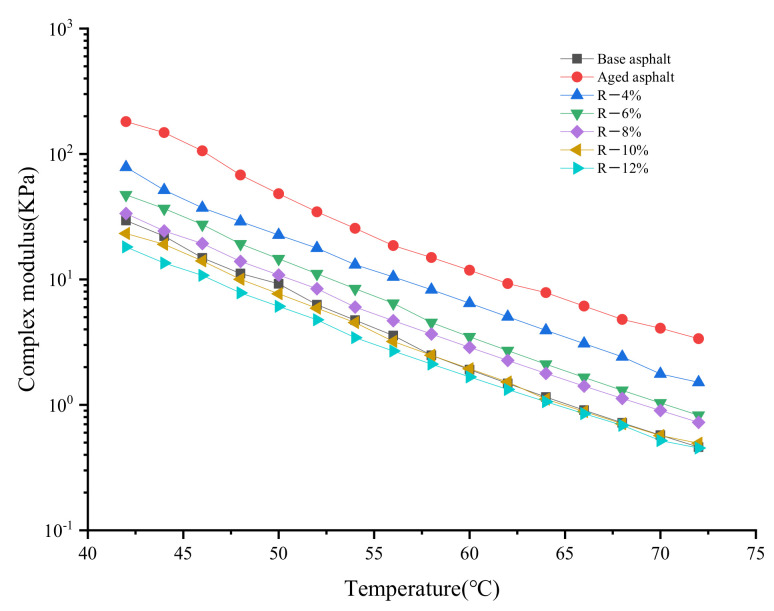
Test results of complex modulus ($G^{*}$) of aged asphalt after the regeneration.

**Figure 2:**
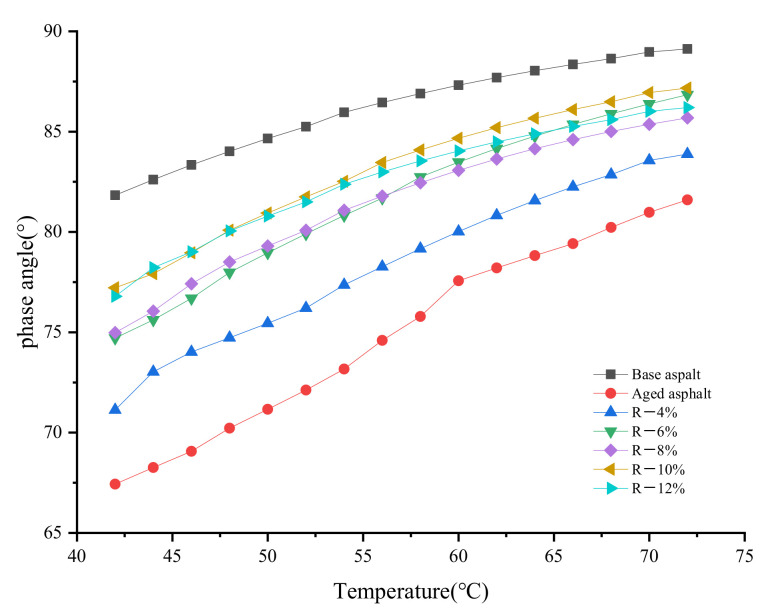
Test results of phase angle (δ) of aged asphalt after the regeneration.

Figure 1 and Figure 2 show the effect of the tung oil composite regenerating agent on the complex modulus and phase angle of aged asphalt. The range of the test temperature is 42–72 °C, and its increase rate is 2 °C/min; the loading frequency ω is 10 rad/s, and the strain control is 12%.

It can be seen from Figure 1 that the complex modulus G* of all asphalt samples decreases gradually with the increase in temperature. For instance, at the initial test temperature, the G* of aged asphalt is nearly 6 times higher than that of base asphalt, indicating that the aging makes the asphalt harder. The addition of the tung oil composite regenerating agent can reduce the complex modulus of reclaimed asphalt, because the tung oil can dissolve macromolecular substances and supplement the light components of asphalt, softening the asphalt and reducing the complex modulus. As the content of the tung oil composite regenerating agent increases, the G* of each reclaimed asphalt gradually decreases, which has a negative influence on the deformation resistance of the asphalt. However, the appropriate content of the tung oil composite regenerating agent can restore the fluidity of the aged asphalt. The G* of the R-8% reclaimed asphalt is close to or even higher than that of base asphalt, partly because the C9 petroleum resin in the composite regenerating agent is favorable to high temperatures. YAN [22] et al. used tung oil as a regenerating agent to restore the high-temperature rheological properties of aged asphalt only to the level of base asphalt. However, the composite regenerating agent of tung oil in this paper caused the high-temperature performance of R-8% asphalt to be better than that of matrix asphalt. Therefore, the tung oil composite regenerating agent can restore and improve the deformation resistance of aged asphalt.

#### 4.2.2. Phase Angle

In Figure 2, it is shown that after the asphalt is aged, the phase angle *δ* decreases and the deformation resistance increases. With the addition of the tung oil composite regenerating agent, the phase angle *δ* of reclaimed asphalt gradually decreases and is still smaller than that of base asphalt, indicating the elastic recovery ability of reclaimed asphalt is better than that of base asphalt.

#### 4.2.3. Rutting Factor

**Figure 3:**
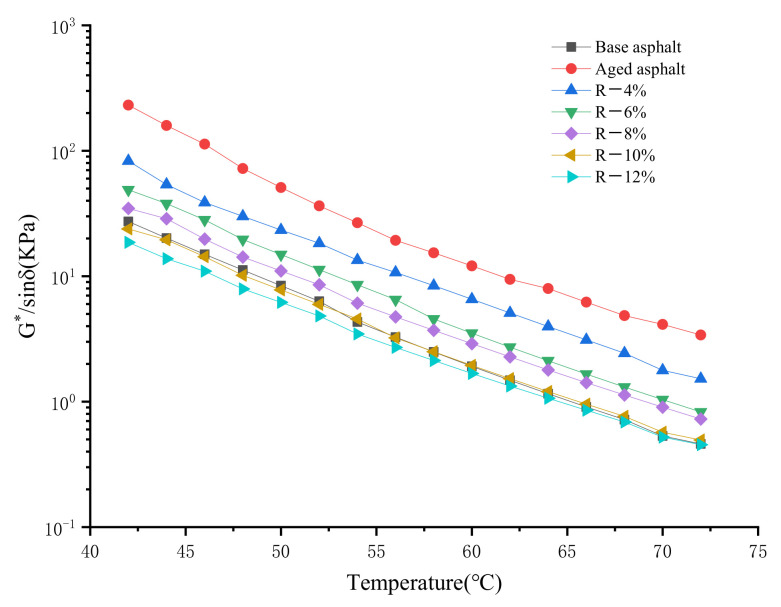
Test results of rutting factor ($G^{*}/\sin\delta$).

The test results of the rutting factor of reclaimed asphalt are shown in Figure 3. As the temperature increases, the $G^{*}/\sin\delta$ of all asphalts gradually decreases. Moreover, as the content of the tung oil composite regenerating agent increases, it also declines, indicating that the addition of the regenerating agent and the increase in temperature reduce the deformation resistance of the asphalt. The $G^{*}/\sin\delta \;$of aged asphalt is the largest, indicating its rutting resistance is the best. As the content of the tung oil composite regenerating agent increases, the $G^{*}/\sin\delta$ of reclaimed asphalt gradually decreases and is close to that of base asphalt. The addition of too much of the tung oil composite regenerating agent can lead to poorer rutting resistance of reclaimed asphalt. Therefore, the proper content of the regenerating agent ensures that the reclaimed asphalt has sufficient rutting resistance. The $G^{*}/\sin\delta$ of R-8% reclaimed asphalt is very close to or even better than that of base asphalt. Thus, the content of the tung oil composite regenerating agent should not exceed 8%.

#### 4.2.4. Master Curve

**Figure 4:**
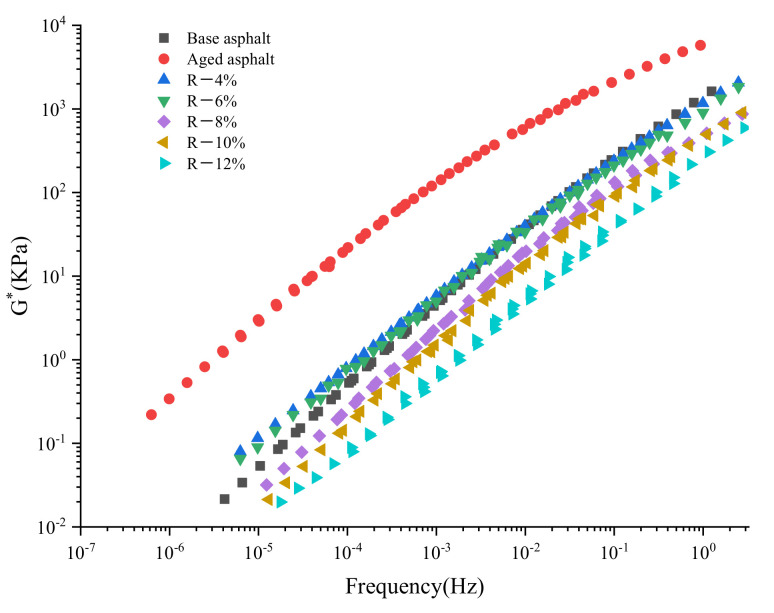
Master curve of complex modulus of reclaimed asphalt.

**Figure 5:**
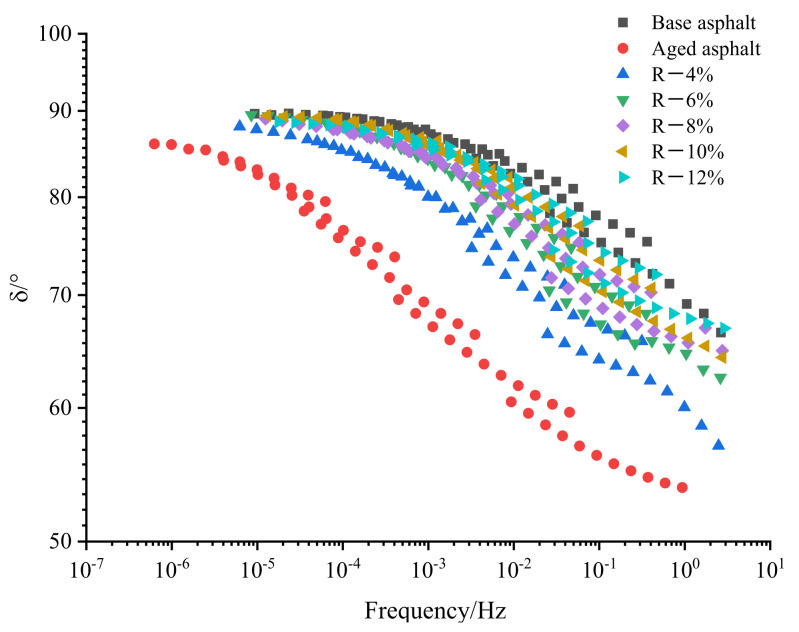
Master curve of phase angle of reclaimed asphalt.

The temperature range of the frequency sweep test is 28–76 °C (the temperature interval is 12 °C), and its sweep frequency is 0.1–10 Hz. According to the WLF equation [26], the master curve of the complex modulus and phase angle constructed at the reference temperature of 20 °C is shown in Figure 4 and Figure 5.

As shown in Figure 4, compared with the base asphalt, the aged asphalt shows a higher complex modulus, which is beneficial to the rutting resistance of RAP at a low frequency and high temperature; G* has a great linear relationship with the frequency. As the content of the tung oil composite regenerating agent increases, the G* of the asphalt shifts close to that of base asphalt and increases with the increase in frequency, which means that the asphalt has the advantage of road deformation resistance at a high-frequency state and a low temperature. As the loading frequency decreases and the temperature increases, the G* of reclaimed asphalt decreases and the G* of R-8% reclaimed asphalt is the closest to that of base asphalt.

It can be seen from Figure 5 that the aged asphalt has the smallest *δ* due to the loss of light components, which increases the proportion of elastic components in the asphalt; the addition of the tung oil composite regenerating agent can increase the *δ* of aged asphalt. As the content of the tung oil composite regenerating agent increases, the *δ* of the asphalt gradually increases and is close to that of base asphalt, indicating that the tung oil composite regenerating agent can increase the proportion of viscous components in the aged asphalt and improve the viscoelastic properties of aged asphalt; Moreover, when the content of the tung oil composite regenerating agent is 8%, the *δ* of R-8% asphalt is smaller than that of base asphalt, indicating that the elastic recovery performance of R-8% asphalt is better than that of base asphalt.

**Figure 6:**
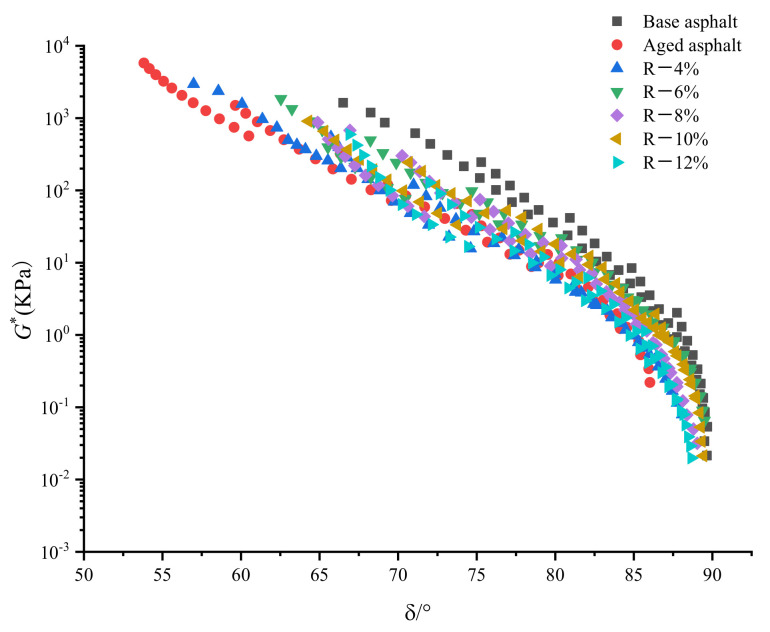
Black diagram of reclaimed asphalt.

The black diagram used to evaluate the viscoelastic properties of asphalt is a diagram of rheological data for asphalt materials in the form of complex modulus and phase angle. Figure 6 shows a black diagram of base asphalt, aged asphalt, and reclaimed asphalt. The curve of aged asphalt is incoherent in the black diagram. However, because the tung oil composite regenerating agent can improve the molecular conformation of aged asphalt to a certain extent, the curve of reclaimed asphalt is smooth, which is basically a coherent curve. The phase angle of reclaimed asphalt is smaller than that of the base asphalt, indicating that the elastic response of reclaimed asphalt is stronger.

#### 4.2.5. Creep Stiffness and Creep Rate

**Figure 7:**
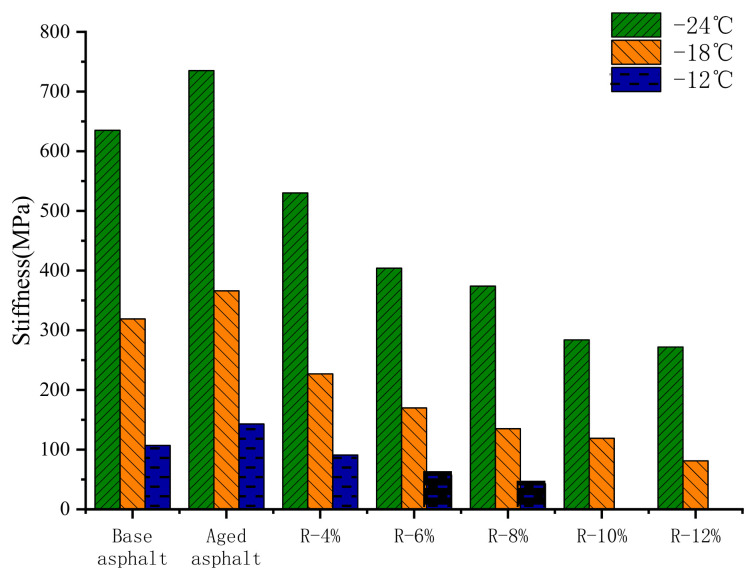
S of different reclaimed asphalt samples.

**Figure 8:**
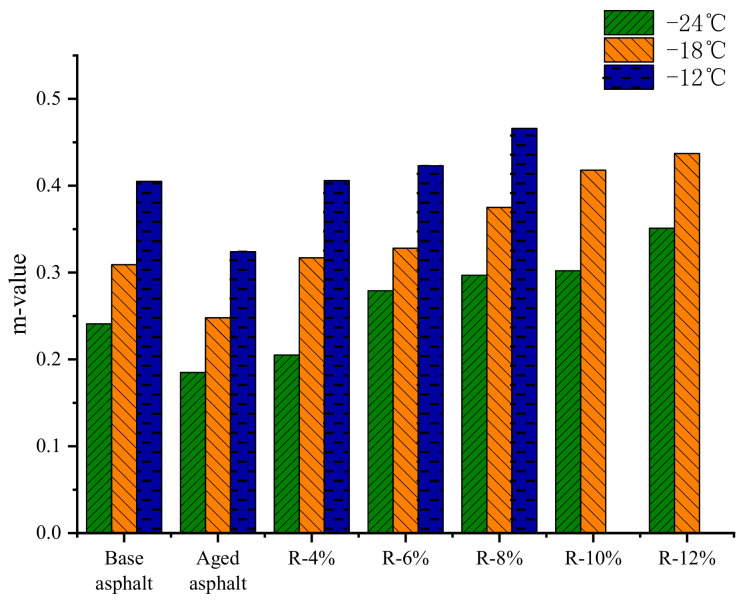
m of different reclaimed asphalt samples.

The results of creep stiffness *S* and creep rate *m* of different asphalt samples were shown in Figure 7 and Figure 8. The test temperatures are −12 °C, −18 °C, and −24 °C. There is no test result for R-10% and R-12% reclaimed asphalts due to their excessive deformation at −12 °C.

It can be seen from Figure 7 and Figure 8 that as the content of the tung oil composite regenerating agent increases, the *S* and *m* of reclaimed asphalt gradually decreases and increases, respectively, indicating that with the addition of the tung oil composite regenerating agent, the low-temperature flexibility and cracking resistance of reclaimed asphalt are gradually improved. YAN [22] restored the low-temperature performance of aged asphalt by using tung oil as a regenerating agent. At −18 °C, the S of the regenerated asphalt with 8% tung oil is 170 MPa, and its m is 0.38, while the content of the tung oil composite regenerating agent in this paper is 8%, the S of R-8% asphalt is 135 MPa, and its m is 0.375, indicating that the tung oil composite regenerating agent has better recovery ability compared to the low-temperature performance of aged asphalt. This is because the plasticizer in the tung oil composite regenerating agent can improve the flexibility, low-temperature ductility, and crack resistance of the asphalt. Compared with those of base asphalt, the *S* and *m* of R-8% reclaimed asphalt decrease by 60% and increase by 15.1% at −12 °C, respectively; at −18 °C, the *S* and *m* decrease by 57.7% and increase by 21.4%, respectively; and at −24 °C, the *S* and *m* decrease by 41.1% and increase by 23.2%, indicating that the tung oil composite regenerating agent can not only restore the *S* and *m* of aged asphalt to the level of base asphalt, but also improve the low-temperature crack resistance of reclaimed asphalt with the optimal content, which is better than that of base asphalt.

### 4.3. Anti-Aging Performance of Reclaimed Asphalt with Composite Regenerating Agent

#### 4.3.1. Thermo-Oxidative Aging Resistance

**Figure 9:**
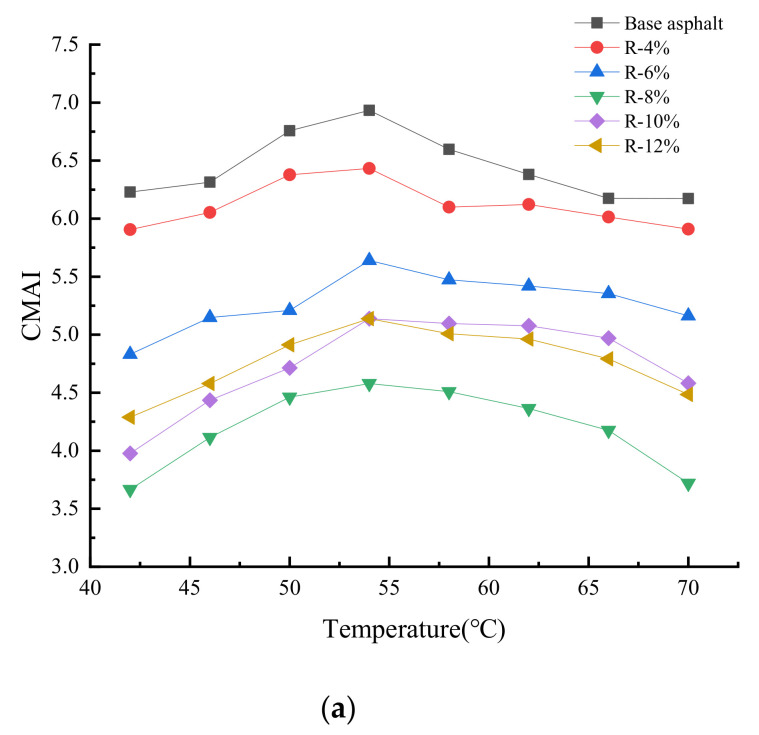

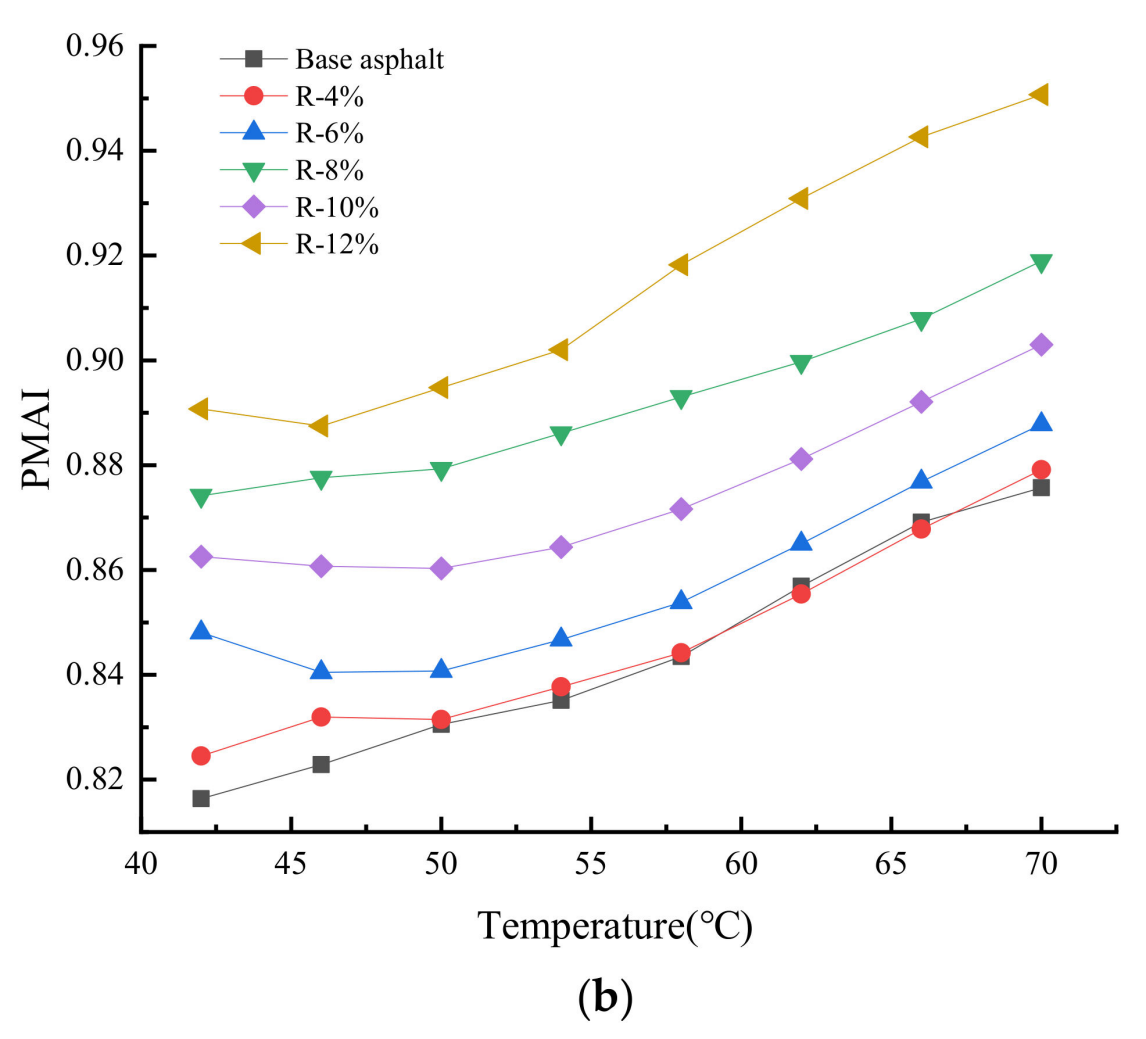
Thermo-oxidative aging resistance of reclaimed asphalt after PAV aging. (**a**) CMAI. (**b**) PMAI.

Figure 9 shows the change trends of CMAI and PMAI of base asphalt and different contents of reclaimed asphalt after the PAV aging. The CMAI of asphalt first increases and then decreases with the increase in temperature. The motion state of asphalt molecules is closely related to the ambient temperature. According to its deformation characteristics under the action of external force, the asphalt can be divided into a glass state, high elastic state, and viscous flow state. Liquids with lower molecular weights usually tend to show lower viscous flow temperatures [27]. Prior to asphalt aging, more light components and fewer heavy components are generated in the asphalt. Under the action of external force, the relaxation time of asphalt molecules is shorter so that the asphalt is more prone to viscous flow, and its viscous flow temperature is relatively low; however, after thermal-oxidative aging, light components decrease and heavy components increase in the asphalt, which leads to an increase in the average relative molecular mass of the asphalt and increases the relaxation time of asphalt molecules; that is, the viscous flow temperature becomes higher. During the temperature scanning process, as the temperature increases, the viscous flow of original asphalt appears earlier than that of aging asphalt at the stage of 42~54 °C so that the complex modulus of original asphalt decays much faster than that of aged asphalt and improves the CMAI of the asphalt. It can be seen from Figure 9a that the CMAI of reclaimed asphalt after PAV aging is smaller than that of base asphalt, indicating that the OMMT in the tung oil composite regenerating agent can effectively block the penetration and propagation of gaseous substances in the asphalt, such as water molecules and oxygen, and delay the aging process of asphalt under thermal-oxidative action. Therefore, the tung oil composite regenerating agent can effectively improve the thermal-oxidative aging resistance of aged asphalt.

As the content of the tung oil composite regenerating agent increases, the CMAI of reclaimed asphalt first decreases and then increases, of which the CMAI of R-8% reclaimed asphalt is the smallest, indicating that the reclaimed asphalt has the best thermal-oxidative aging resistance. When the content of the tung oil composite regenerating agent exceeds 8%, the CMAI of reclaimed asphalt gradually increases. Thus, when the content of the tung oil composite regenerating agent is 8%, the reclaimed asphalt can be guaranteed to have better thermal-oxidative aging resistance. It can be seen from Figure 9b that the phase angle of the asphalt decreases after PAV aging, and more viscous components in the asphalt are transformed into elastic components; when the content of the tung oil composite regenerating agent is 4%, the PMAI of reclaimed asphalt is close to that of base asphalt; and when its content exceeds 4%, the PMAI of reclaimed asphalt is larger than that of base asphalt, indicating that the tung oil composite regenerating agent can restore the thermo-oxidative aging resistance of aged asphalt and effectively improve the thermo-oxidative aging resistance of reclaimed asphalt.

#### 4.3.2. UV Aging Resistance

**Figure 10:**
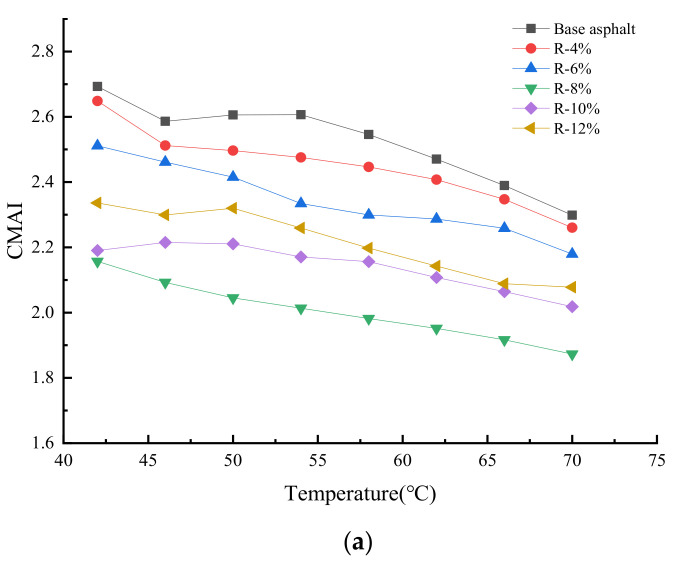

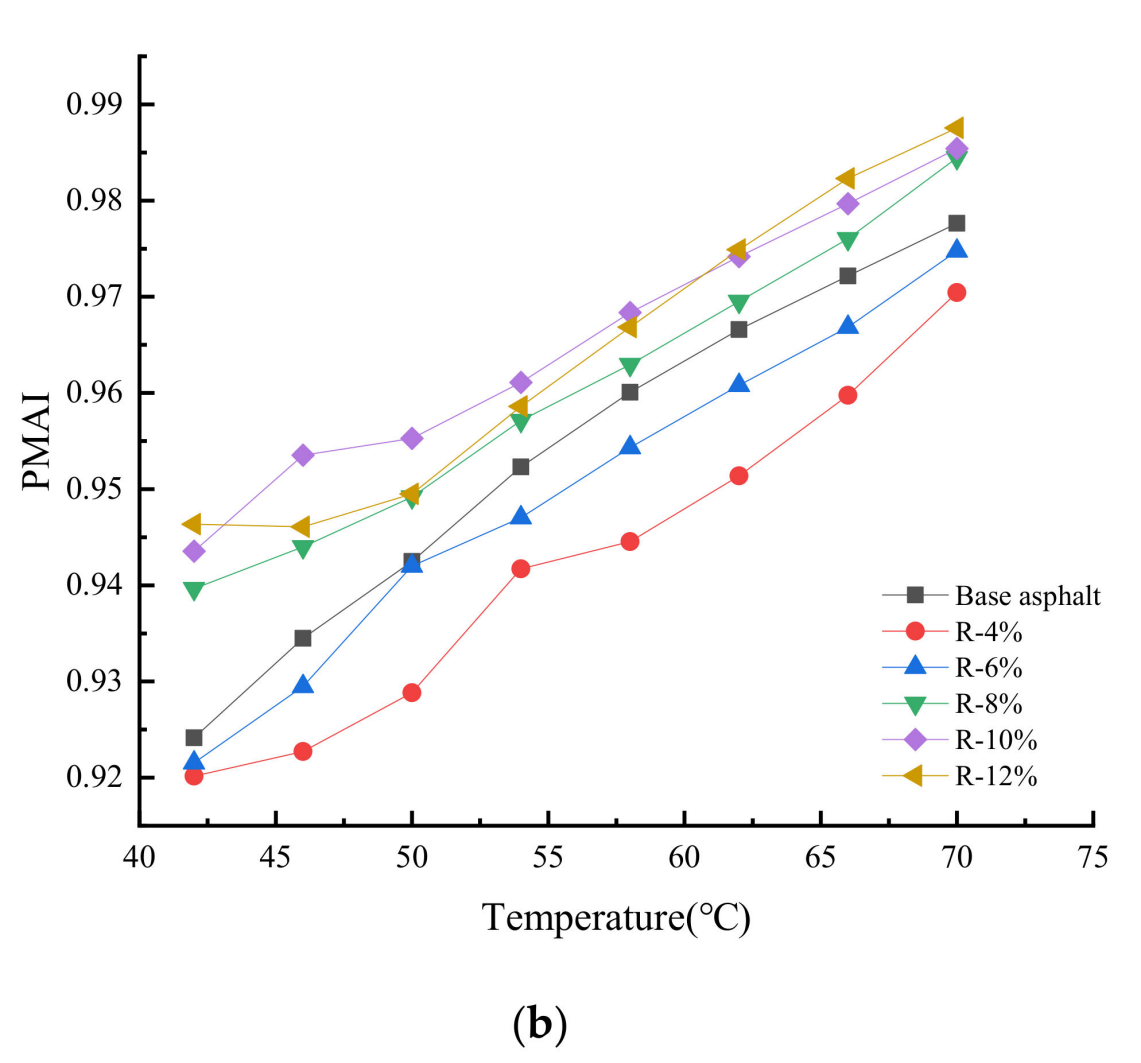
UV aging resistance of reclaimed asphalt after the UV aging. (**a**) CMAI. (**b**) PMAI.

In Figure 10, the change trends of CMAI and PMAI of base asphalt and reclaimed asphalt with different contents after UV aging are shown. It can be seen from Figure 10a that the CMAI of base asphalt ranges from 2.2 to 2.7 and gradually decreases with the increase in temperature; and the CMAI of reclaimed asphalt is lower than that of base asphalt, indicating that the tung oil composite regenerating agent can reflect and absorb the UV light and decrease the damage of UV light to the reclaimed asphalt. When the content of the tung oil composite regenerating agent increases to 8%, the CMAI of reclaimed asphalt is the smallest, and thereby its UV aging resistance is the best. As shown in Figure 10b, the PMAI of base asphalt is 0.92~0.98 and increases linearly with the increase in temperature, while the PMAI of R-8%, R-10%, and R-12% reclaimed asphalts is larger than that of base asphalt, indicating that the tung oil composite regenerating agent can improve the UV aging performance of reclaimed asphalt. However, when the content of the tung oil composite regenerating agent exceeds 8%, the PMAI of reclaimed asphalt tends to decrease. As a result, when the content of the tung oil composite regenerating agent is 8%, it can be ensured that the reclaimed asphalt has better UV aging resistance.

### 4.4. Microstructure and Mechanism Analysis of Reclaimed Asphalt with Composite Regenerating Agent

#### 4.4.1. Morphology

**Figure 11:**
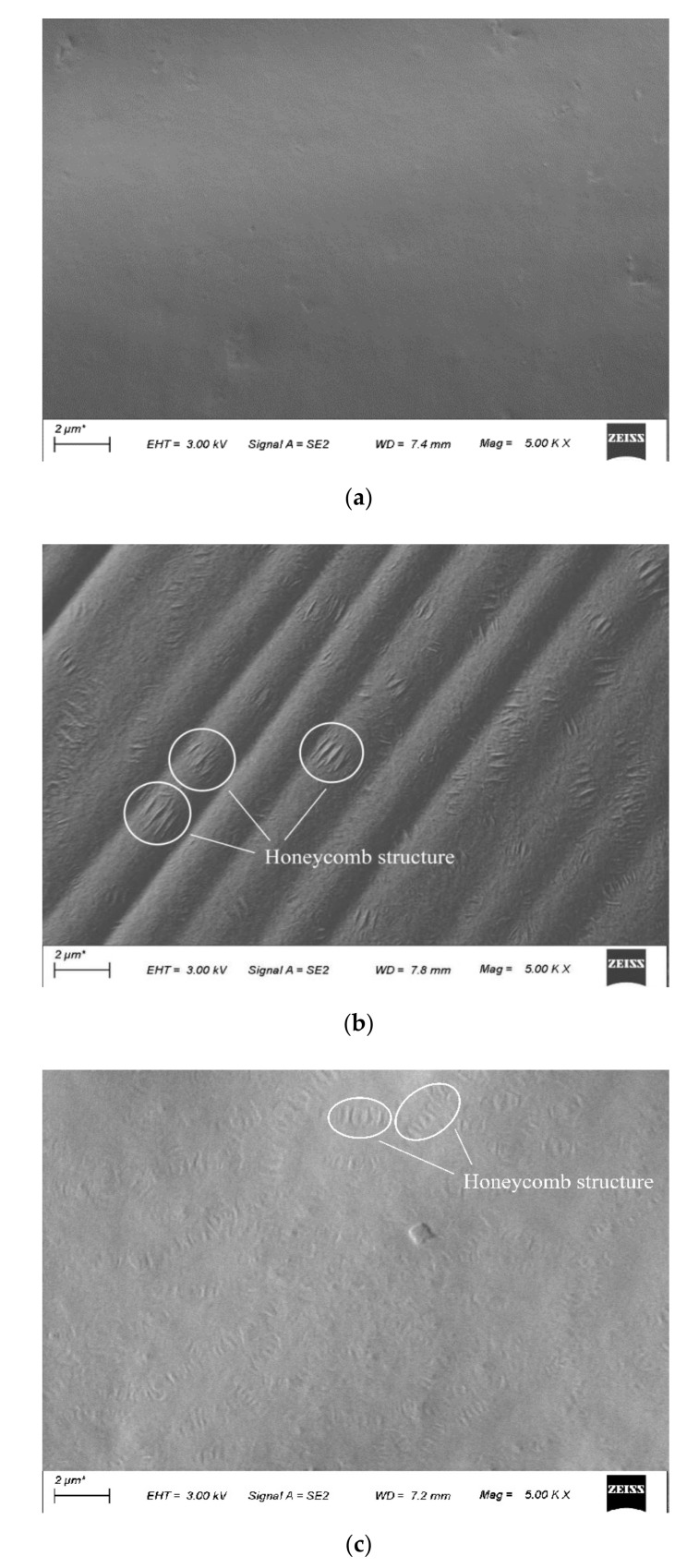
Micromorphologies of different asphalts under SEM (5000 times). (**a**) Base asphalt. (**b**) Aged asphalt. (**c**) R-8%.

A Zeiss sigma 300 SEM was used to collect the surficial micro-morphologies of asphalt samples. The microstructures and morphologies of base asphalt, aged asphalt, and reclaimed asphalt are shown in Figure 11. It can be seen from Figure 11 that the overall surface of base asphalt is in a flat and smooth state, which is basically a homogeneous structure, and a large number of wrinkles appear on the surface of aged asphalt. This is because the light components decrease, the molecular polarity increases, the molecular movement ability is weakened, and the asphalt fluidity deteriorates after aging. With the addition of the tung oil composite regenerating agent, the surface of R-8% reclaimed asphalt tends to be flat and smooth, which is similar to that of base asphalt. In addition, the uneven striped “honeycomb structure” can be seen from the graphs of both aged asphalt and reclaimed asphalt, and the area of a single honeycomb structure of aged asphalt is larger than that of reclaimed asphalt, which may be due to the aggregation of macromolecular asphaltenes of the asphalt [20]. After the tung oil composite regenerating agent is added, the light components increase. Furthermore, the area of the honeycomb structure of R-8% reclaimed asphalt decreases, and its surface tends to be smooth, indicating that the tung oil composite regenerating agent can roughly restore the microstructure and morphology of aged asphalt.

#### 4.4.2. Composition of Regenerating Agent and Reclaimed Asphalt

**Figure 12:**
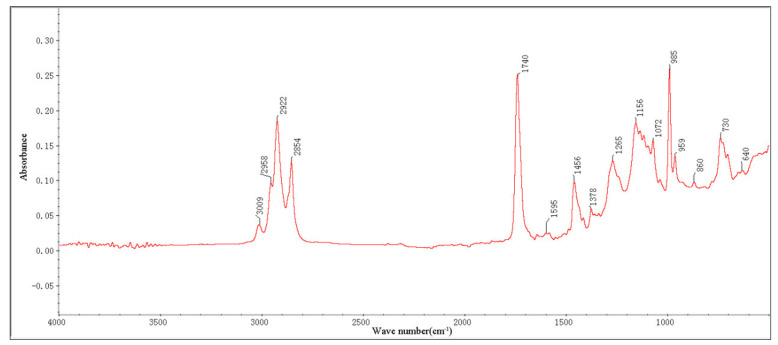
Infrared spectrum of tung oil composite regenerating agent.

**Figure 13:**
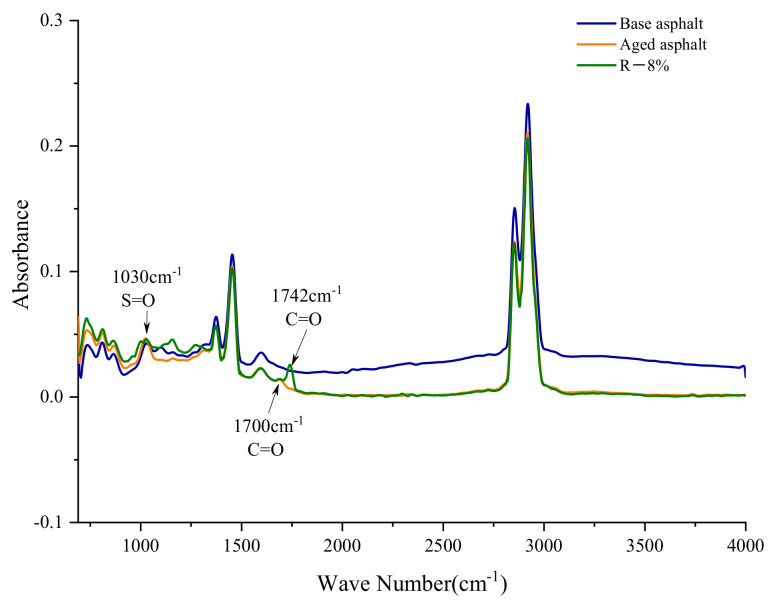
FTIR images of different asphalt samples.

The functional groups of the asphalt samples were determined by the Nicolet iS50 FTIR spectrometer. The wavelength range of the test is 500–4000 cm^−1^, and the number of scans is 32. The infrared spectra are shown in Figure 12 and Figure 13.

The infrared spectrum of the tung oil composite regenerating agent is shown in Figure 12. Through the analysis of characteristic peaks in the infrared spectrum of the tung oil composite regenerating agent, it is found that the absorption peak near 2958 cm^−1^ is a CH_3_ antisymmetric and symmetric stretching vibration, while the absorption peak in the interval of 2922 cm^−1^~2854 cm^−1^ is a -CH_2_ antisymmetric and symmetric stretching vibration, indicating that the tung oil composite regenerating agent contains non-polar methyl and methylene functional groups. The absorption peak near 3009 cm^−1^ is the C-H stretching vibration. The tung oil composite regenerating agent has absorption peaks of C=C stretching vibration of three aromatics near 1595 cm^−1^, 1456 cm^−1^, and 1378 cm^−1^, and C-H bending vibration of the benzene ring in the interval of 900 cm^−1^~650 cm^−1^, indicating that the main components of the tung oil composite regenerating agent are light components rich in aromatic hydrocarbons. There is a C=O stretching vibration absorption peak of saturated fatty acid ester near 1740 cm^−1^ and a C-O stretching vibration absorption peak near 1265 cm^−1^, 1156 cm^−1^, and 1072 cm^−1^. Moreover, there is a C-H in-plane bending and stretching characteristic peak in the interval of 900 cm^−1^~600 cm^−1^, indicating that the tung oil composite regenerating agent is rich in aromatic compounds and has good compatibility with the asphalt.

Figure 13 shows the infrared spectra of base asphalt, aged asphalt, and reclaimed asphalt. It can be found from Figure 13 that the positions of characteristic peaks of all asphalts are almost the same. Compared with the base asphalt, the aging asphalt has an absorption peak caused by the carbonyl C=O at 1700 cm^−1^, and the characteristic peak of sulfoxide group S=O at 1030 cm^−1^ increases, which is caused by the oxidation reaction during the aging process of the asphalt. For the reclaimed asphalt, a new characteristic peak appears near 1742 cm^−1^ after the addition of the tung oil composite regenerating agent, which is caused by the C=O stretching vibration of saturated fatty acid ester. In addition, no other characteristic peaks appear in the reclaimed asphalt. Its characteristic peak is almost the same as that of base asphalt, indicating that the tung oil composite regenerating agent has no chemical reaction with the asphalt, rather only physical blending.

#### 4.4.3. Molecular Weight and Distribution of Different Asphalts

The molecular weight and distribution of asphalt were analyzed by Waters 1515 GPC, and tetrahydrofuran (THF) was used as the mobile phase. The concentration and flow rate of the asphalt sample are 2 mg/mL and 10 mL/min, respectively.

**Figure 14:**
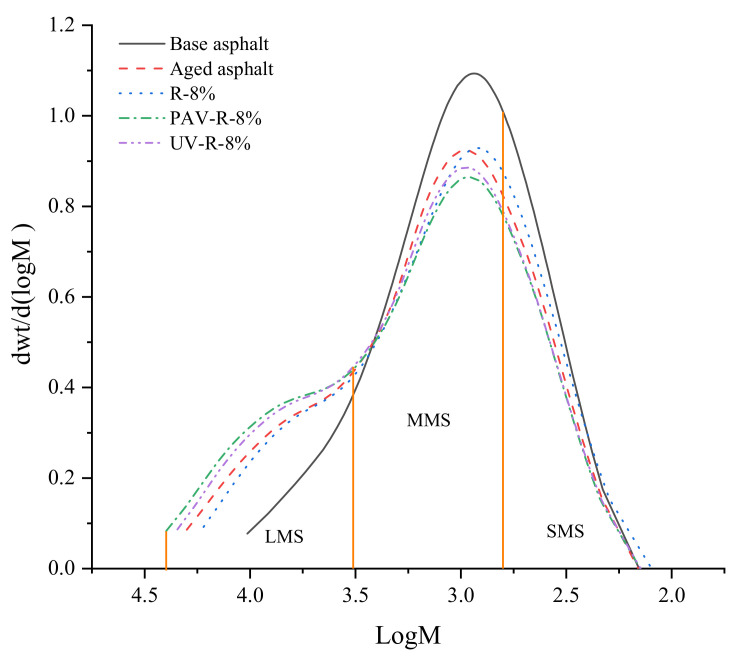
Molecular weight distribution of different asphalts.

**Figure 15:**
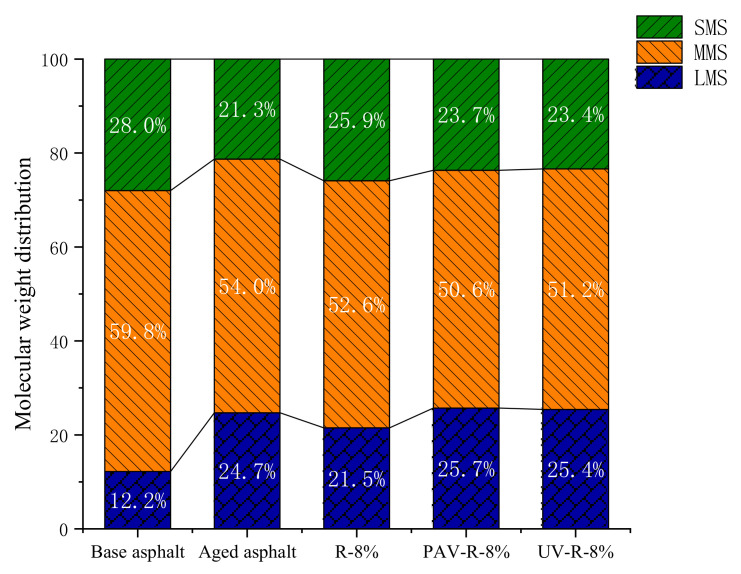
Molecular weight proportion of different asphalts.

In Figure 14, the abscissa of the GPC curve is the molecular weight, and its ordinate is the differential distribution of molecular weight. The GPC is usually divided into 13 blocks, of which blocks 1–5 are macromolecules (LMS), blocks 6–9 are medium molecules (MMS), and blocks 10–13 are small molecules [28]. According to the distribution of molecular weight in Figure 14, the integral areas of LMS, MMS, and SMS of base asphalt, aged asphalt, and reclaimed asphalt were calculated, and the content of each molecule of LMS, MMS, and SMS was obtained, as shown in Figure 15. It can be seen from the figure that compared with those of base asphalt, the LMS content of aged asphalt increases by 12.5%, while the MMS and SMS contents decrease by 5.8% and 6.7%, respectively. This is because, in the process of thermo-oxidative aging, there is a polymerization reaction between aromatic and colloidal components of small molecular weight and asphaltenes of a large molecular weight produced, thereby enhancing the intermolecular force of the asphalt and weakening its molecular movement ability. Compared with those of aged asphalt, the contents of LMS and MMS of R-8% reclaimed asphalt decrease by 3.2% and 1.4%, respectively, and the content of its SMS increases by 4.6%. It is shown that the tung oil composite regenerating agent contains a certain number of medium and small molecules, which can fully supplement small and medium molecules in the components of aging asphalt and dissolve a small part of the macromolecules, thus solving the agglomeration problem of macromolecules.

In Figure 15, it is shown that after the PAV and UV aging, the LMS and MMS of R-8% reclaimed asphalt have different increasing trends, while its SMS has decreasing trends. Furthermore, the molecular weight of PAV-aged asphalt is larger than that of UV-aged asphalt, indicating that during the UV aging process, the R-8% reclaimed asphalt without thermo-oxidative aging loses few medium and small molecules. According to the change trend of the molecular weight of LMS, MMS, and SMS in Figure 15, the change range of molecular weight of base asphalt and R-8% reclaimed asphalt after the PAV aging was calculated, as shown in Table 6.

It can be seen from Table 6 that after PAV aging, the LMS content of base asphalt increases by 12.5%, while its MMS and SMS contents decrease by 5.8% and 6.7%, respectively. Furthermore, the LMS content of R-8% reclaimed asphalt increases by 4.2%, while its MMS and SMS contents decrease by 2% and 2.2%, respectively. It is obvious that the change range of the molecular weight of R-8% reclaimed asphalt is small, indicating that the aging performance of R-8% reclaimed asphalt decays slowly after PAV aging, which is beneficial to the aging resistance of reclaimed asphalt. Moreover, it can be found from Figure 15 that after PAV aging, the SMS content of R-8% reclaimed asphalt is more than that of base asphalt, indicating that the tung oil composite regenerating agent can inhibit the loss of small molecules in the reclaimed asphalt.

## 5. Conclusions

- The optimal mix proportion of the tung oil composite regenerating agent was determined by the orthogonal design test method; that is, tung oil: DOP: C9 petroleum resin: OMMT = 25:5:2:3.
- As the content of the tung oil composite regenerating agent increases, the rutting factor and creep stiffness gradually decrease and the creep rate increases, indicating that the tung oil composite regenerating agent can restore the rheological properties of aged asphalt, which is even better than that of base asphalt. The CMAI of reclaimed asphalt is smaller than that of base asphalt, while the PMAI of reclaimed asphalt is larger than that of base asphalt. The anti-aging ability of reclaimed asphalt is significantly improved, and the optimal content of the tung oil composite regenerating agent is 8%.
- As the content of macromolecules increases, the fluidity of aged asphalt becomes poor and a wrinkled texture and large honeycomb structure appear on its surface. The addition of the tung oil composite regenerating agent can restore the morphological features of aged asphalt to a certain extent, which makes its surface tend to be flat and smooth, and the size of te honeycomb structure is reduced.
- The FTIR diagram shows that the tung oil composite regenerating agent is mainly composed of light components rich in aromatic hydrocarbons, and the characteristic peaks of reclaimed asphalt are basically consistent with those of base asphalt, indicating that the tung oil composite regenerating agent is beneficial to the dispersion and dissolution of polar substances in the aged asphalt.
- The GPC results of reclaimed asphalt show that the tung oil composite regenerating agent can reduce the content of macromolecule in the aged asphalt, and the change range of molecular weight of reclaimed asphalt after aging is smaller than that of base asphalt, indicating that the aging of reclaimed asphalt decays slowly, which is favorable for the aging resistance of reclaimed asphalt.

## Figures and Tables

**Table 1:** Technical indexes of tung oil.

Raw Materials	Appearance	Density/g·cm^−1^	Flash Point/°C
Tung oil	Yellow liquid	0.943	236
DOP	Colorless oily liquid	0.985	225
C9 petroleum resin	Yellow particle	0.995	260
OMMT	White powder	1.03	-

**Table 2:** Technical indexes of substrate asphalt and aged asphalt.

Technical Indexes	Base Asphalt	Aged Asphalt	Test Methods
Penetration (25 °C)/0.1 mm	68.7	21.1	ASTM D5
Ductility (15 °C)/cm	142.0	5.6	ASTM D113
Softening point (ring and ball method)/°C	48.0	65.6	ASTM D36
Viscosity (135 °C)/mPa∙s	485	936	ASTM D4402

**Table 3:** Factor levels of orthogonal test design.

Levels	Tung Oil (A)/%	DOP (B)/%	C9 Petroleum Resin (C)/%	OMMT (D)/%
Level 1	75	10	14	1
Level 2	70	15	10	5
Level 3	65	20	6	9

**Table 4:** Orthogonal test results.

No.	Tung Oil (A)/%	DOP(B)/%	C9 Petroleum Resin (C)/%	OMMT(D)/%	Softening Point/°C	Penetration/0.1 mm	Ductility/cm	Viscosity/mPa∙s
1	75	10	14	1	48.4	93.5	143.6	480.0
2	75	15	10	5	47.0	100.1	138.2	455.0
3	75	20	6	9	45.1	130.0	142.6	404.0
4	70	10	10	9	46.6	145.2	125.4	447.0
5	70	15	6	1	46.7	103.2	134.3	426.0
6	70	20	14	5	48.4	82.5	121.0	477.5
7	65	10	6	5	46.5	99.6	117.4	451.0
8	65	15	14	9	47.2	98.9	119.3	483.0
9	65	20	10	1	48.2	86.0	97.6	505.0

**Table 5:** Preferred combinations of orthogonal tests.

Indexes	Preferred Combinations
Softening point/°C	A_1_B_2_C_3_D_3_
Penetration/(0.1mm)	A_2_B_2_C_3_D_3_
Ductility/cm	A_1_B_2_C_3_D_3_
Viscosity/(mPa∙s)	A_1_B_2_C_3_D_3_

**Table 6:** Change range of molecular weight of asphalt after PAV aging.

Types of Asphalt	LMS (%)	MMS (%)	SMS (%)
Base asphalt	12.5	−5.8	−6.7
R-8% reclaimed asphalt	4.2	−2	−2.2

## Data Availability

The data presented in this study are available on request from the corresponding author.
